# Hurler disease (mucopolysaccharidosis type IH): clinical features and consanguinity in Tunisian population

**DOI:** 10.1186/1746-1596-6-113

**Published:** 2011-11-10

**Authors:** Latifa Chkioua, Souhir Khedhiri, Hadhami Ben Turkia, Henda Chahed, Salima Ferchichi, Marie Françoise Ben Dridi, Sandrine Laradi, Abdelhedi Miled

**Affiliations:** 1 Laboratory of Biochemistry Farhat Hached Hospital 4000 Sousse - Tunisia; 2 Laboratory of Molecular Biology University of Pharmacy 5000 Monastir - Tunisia; 3 Laboratory of Pediatric La Rabta Hospital Tunis-Tunisia

**Keywords:** mucopolysaccharidosis type I, Tunisian population, consanguinity, mutations

## Abstract

Mucopolysaccharidosis type I (MPS I) was a group of rare autosomal recessive disorder caused by the deficiency of the lysosomal enzyme, alpha -L -iduronidase, and the resulting accumulation of undergraded dematan sulfate and heparan sulfate. MPS I patients have a wide range of clinical presentations, that makes it difficult to predict patient phenotype which is needed for genetic counseling and also impedes the selection and evaluation of patients undergoing therapy bone marrow transplantation.

**Aim of the study:**

consanguinity rates have been determined among 14 families with mucopolysaccharidosis type I, seen in the pediatric departments of different geographic areas of Tunisia (Central and Southern areas) for the period August 2004 - August 2011 in order to investigate the relation between consanguinity and this disorder.

**Patients and methods:**

Clinical and molecular analyses confirmed the diagnosis for MPS type I in the studied families.

**Results:**

Most of the Tunisian MPS I patients have been identified at the homozygous status: p.P533R mutation (7 homozygous and one double heterozygous p.L578Q/p.P533R patients; 41.66% of all the investigated MPSI patients), p.F177S (1 homozygous patient; 5.55%), p.L530fs (1 patient; 5.55%), p.Y581X (2 patients; 11.11%), p.F602X (3 patients; 16.66%), p.R628X (1 patient; 5.55%). Another mutation: p.L578Q has been identified at the heterozygous status in the only double heterozygous p.L578Q/p.P533R case. Part of the mutations was the result of a founder effect. These described points are the consequences of the high rate of consanguinity.

**Conclusion:**

The high frequency of p.P533R mutation could be explained by the high degree of inbreeding. This is due to the richness of the genetic background of the studied population.

A multidisciplinary approach is essential to develop adequate preventive program adapted to the social, cultural, and economic context.

## Background

Mucopolysaccharidoses (MPS) are a group of lysosomal storage and inherited disorders caused by the deficiency of specific enzymes which leads to the lysosomal accumulation of glycosaminoglycanes. Mucopolysaccharidosis type I is caused by a deficiency of the glycosidase alpha-L-iduronidase (α-L-iduronidase iduronohydrolase, EC 3.2.1.76; IDUA) and the resulting accumulation of undergraded dermatan sulfate and heparan sulfate [1]. Accumulation of partially degraded of this macromolecules leads to typical symptoms of lysosomal storage disorder. Clinical phenotypes may vary considerably so that 3 different forms, severe (MPS IH, Hurler), mild (MPS IS, Scheie), and intermediate (MPS IH/S, Hurler/Scheie) are individualized.

The most severe form as well as the most common and the best known of the Hurler syndrome is characterized by retardation of physical and mental development, cornea clouding, dysostosis multiplex, joint stiffness, cardiovascular involvement, respiratory problems, and death in childhood. The mildest form (Scheie syndrome), is compatible with normal intelligence, stature and lifespan; this form of disease progresses slowly with cloudy corneas, joint stiffness and aortic valve disease as the major problems. The intermediate form (Hurler/Scheie syndrome) is characterized by normal intelligence with physical manifestations intermediate between Hurler and Scheie syndromes. Survival commonly occurs at early adulthood.

Tunisia is one of the North African countries, geographically situated in a central position at the crossroad between Africa and Europe. The demographic features of the Tunisian population include among others high rates of consanguinity.

Consanguineous marriages are associated with a higher frequency of autosomal recessive disorders and more precisely between first cousins [2]. This rule results from particular traditions as a consequence of cultural and economical reasons.

In addition, Arab countries including Tunisia are known to present a high number of mothers over 35 years of age, a large family size, a high frequency of hemoglobinopathies [3] and Cystic fibrosis [4]. Consanguinity still continues to attract attention among medical and population geneticists, clinicians and scientists.

Thus, an association between parental consanguinity and the frequency of MPS IH will be reported and discussed.

### Patients and methods

**Figure 1:**
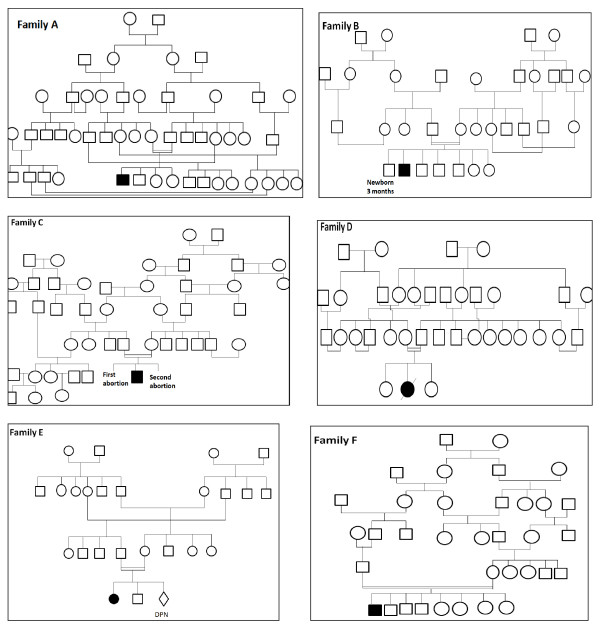
**Pedigrees of the six investigated MPS I Tunisian families**. Squares and circles indicate male and female members, respectively. Double lines indicate consanguineous matings.

We report here on the spectrum of MPS IH diseases in Tunisia. The study was carried out on 14 MPS I families recruited in Pediatric departments of different geographic areas of Tunisia (Central and Southern areas). The phenotypic and molecular diagnoses were confirmed in our laboratory of Biochemistry at the Farhat Hached Hospital of Sousse in Tunisia. This actual study followed the previous work which already included 6 MPS I families (family A to family F) (Figure 1).

### Familial questionnaire

Tunisia is one of the Muslim North African countries, geographically situated in a central position at the crossroad between Africa and Europe. The estimated population in July 2011 is 10,629,186 inhabitants with a population growth rate of 0.978% *(*http://www.indexmundi.com/fr/tunisie/population_profil.html*)*. Nearly all the Tunisians are Muslims (98% of the population) the others are Jewish, Christians. Consanguinity rates were studied among 14 families with mucopolysaccharidosis type I, seen in the Pediatric departments of different geographic areas of Tunisia (Central and Southern areas) for the period August 2004 to August 2011. All the patients are offspring of consanguineous marriages between first, second or third cousins (Figure 1 and Table 1).

**Table 1:** consanguinity profile and genotypes characteristics of MPS I Tunisians patients

MPS I families	age of parents at birth	relationship (affected/unaffected siblings)	origin	Consanguinity	Mutations	MPS I subtype	references
1	38 years (M), 40 years (F)	3/0	Sfax (South)	3^rd ^degree	p.F602X_H_	Hurler	[11]

2	55 years (M), 65 years (F)	2/2	Gabes (South)	1^st ^degree	p.P533R_H_	Hurler	[11]

3	40 years (M), 49 years (F)	2/2	Djerba Island (South)	2^rd ^degree	p.P533R_H_	Hurler/Scheie	[11]

4	34 years (M), 40 years (F)	1/3	Tunis (North)	1^st ^degree	p.P533R_H_	Hurler/Scheie	[11]

5	50 years (M), 62 years (F)	1/6	Mahdia (Center)	1^st ^degree	p.R628X_H_	Hurler	[11]

6	42 years (M), 50 years (F)	1/3	Sousse (Center)	1^st ^degree	p.P533R_H_	Hurler	[11]

7	37 years (M), 44 years (F)	1/6	Djerba Island (South)	2^rd ^degree	p.L578Q/p.P533R	Scheie	[11]

8	38 years (M), 46years (F)	1/1	Makther	2^rd ^degree	p.Y581X_H_	Hurler	[11]

A	42 years (M), 46 years (F)	1/3	Sfax (sud)	3^rd ^degree	p.P533R_H_	Scheie	[12]

B	41 years (M), 41 years (F)	1/6	Djerba Island (South)	2^rd ^degree	p.P533R_H_	Hurler	[12]

C	37 years (M), 44 years (F)	1/0	Bizerte (North)	1^st ^degree	p.L530fs_H_	Hurler	[12]

D	39 years (M), 39 years (F)	1/2	Tunis (North)	1^st ^degree	p.Y581X_H_	Hurler	[12]

E	38 years (M), 42years (F)	1/2	Tunis (North)	1^st ^degree	p.F177S_H_	Hurler	[12]

F	42 years (M), 46 years (F)	1/7	Djerba Island (South)	2^rd ^degree	p.P533R_H_	Hurler	[12]

M: Mother

F: Father

_H: _homozygous status

The families of MPS I patients have been questioned to establish an inventory of the consanguineous unions and to determine the relation between consanguinity and the frequency of MPS I Tunisia. Each studied family accepted our preliminary family inquiry on one hand about for their awareness of the seriousness of the disease and on the other hand, about the intensity of their commitment to hope to find a cure for this disease.

This study was approved by the Ethics committees of the Tunisian hospitals, and all families gave informed consent

## Results

The biological, enzymatic and molecular analyses confirmed the MPS diagnosis for all the studied patients. The clinical characteristics of MPS I patients have been reported below (Table 1).

No major mutations appear to be specific to our country and there is a tremendous heterogeneity in the mutation spectrum. No mutation represents more than 53% of the Tunisian MPS I alleles and in approximately 3% of the alleles the mutations are novel or very rare. Therefore, rarely in one case the genotype of the patient shows a unique combination of mutation (Table 2).

**Table 2:** Mutation frequencies in the MPS I Tunisian patients investigated [2004-2011]

Type of the MPS I Mutation	Number of the MPS I allele for the considered mutation/Total of Tunisian MPS I allele	Percentage of the MPS I allele of the considered mutation
p.P533R	15/28	53.57

p.R628X	2/28	7.14

p.F602X	2/28	7.14

p.Y581X	4/28	14.28

p.L530fs	2/28	7.14

p.F177S	2/28	7.14

p.L578Q	1/28	3.57

### Patients of family A

This boy of 11 years was symptomatic when MPS has been detected in the hospital of Sousse by biological assay with very high urinary excretion of glycosaminoglycans (136 mg/g of creatinine) (usual values: 6-23) and abnormal electrophoresis profile (dermatan sulfate and heparan sulfate). MPS I was confirmed by enzymatic assay with a very low IDUA activity (0.1 nmol/h/mg) (usual values: 1-5). He presented psychomotor retardation: he walked at the age of 1 and 6 months and he speaked at the age of 2 years. His clinical features included coarse facial features, hydrocephalus, hepatosplenomegaly. He developed enlarged tongue, thick lips, bushy eyebrows, exophtalmos, noisy breathing and hearing loss. He had, at 10 years and 10 months, 114 cm (-4 S.D.) of height and 28 kg (-1 S.D.) of weight.

### Patients of family B

At the age of 1 year and 4 months, the boy was referred to Pediatric Unit at Djerba Island Hospital for three days. He developed macrocephaly, hepatosplenomegaly, psychomotor retardation with especially language problems. At admission, the examination highlighted short neck and trunck, spacing and shape teeth, enlarged tonsils, enlarged tongue and thick lips. At the age of three years, he had 98 cm (+2 S.D.) of height and 18 kg (+3 S.D.) of weight.. The biochemical assay showed very high urinary excretion of glycosaminoglycans (120 mg/g of creatinine) (usual values: 9-46) and abnormal electrophoresis profile with dermatan sulfate and heparan sulfate. MPS I was confirmed by enzymatic assay with a very low IDUA activity (0.08 nmol/h/mg) (usual values: 1-5). This patient (3 years old) whose parents were second cousin, had a family history with three maternal cousins: one of them (13 years old) was followed for convulsing encephalopathy and infirmity matrix, the other cousin (3 years old) was followed in the same Pediatric Unit for encephalopathy.

### Patient of family C

This little boy was the first child of first cousin parents. The boy was referred to Pediatric Unit at Bizerte Hospital. At the age of 1 year and 8 months, he had 79 cm (-2 S.D.) of height and 10 kg (+4 S.D.) of weight and he developed psychomotor retardation with abnormal flexibility of joints, facial dysmorphisms with coarse features and dystrophia of cornea. The biological assay showed very high urinary excretion of glycosaminoglycans (116 mg/g of creatinine) (usual values: 9-46) and abnormal electrophoresis profile with dermatan sulfate and heparan sulfate. MPS I was confirmed by enzymatic assay with a very low *IDUA activity *(0.1 nmol/h/mg) (usual values: 1-5). He deceased at the age of two years.

### Patient of family D

This girl, whose parents were first cousin, was diagnosed at the age of 2 months and 5 days. She was referred to Pediatric Unit at La Rabta Hospital. She was evaluated for dyspnea and laryngeal crisis. At the age of 14 months she presented facial dysmorphisms. At the age of 3 years and 6 months she had 90 cm (+3 S.D.) of height and 15 kg (+3.5 S.D.) of weight. At the age of 10 years she had 95 cm (-5 S.D.) of height and 18 kg (-2 S.D.) of weight.. The biological assay showed very high urinary excretion of glycosaminoglycans (97 mg/g of creatinine) (usual values: 9-46) and abnormal electrophoresis profile with dermatan sulfate and heparan sulfate. MPS I was confirmed by enzymatic assay with a very low *IDUA activity *(0.1 nmol/h/mg) (usual values: 1-5)MPS I was confirmed by enzymatic assay with a very low IDUA activity (0.0 nmol/h/mg) (usual values: 1-5).

### Patient of family E

The 2 years girl has been followed up since the age of 18 months for MPS. She was referred to Pediatric Unit at La Rabta Hospital. MPS I was confirmed by enzymatic assay with a very low IDUA activity (0.5 nmol/h/mg) (usual values: 1-5 nmol/h/mg). Gradually, she developed facial dysmorphisms, scaphocephaly, skeletal dysplasia, spacing and shape teeth, enlarged tonsils, thick lips, bushy eyebrows, umbilical hernia, mitral valve regurgitation and coax valga. She had also hepatosplenomegaly and cardiomegaly. Cervical MRI indicated compression of the medulla. At the age of 4 years she had 103 cm (-2.7 S.D.) of height and 18 kg (+2 S.D.) of weight.. The biochemical analysis showed very high urinary excretion of glycosaminoglycans (125 mg/g of creatinine) (usual values: 9-46) and abnormal electrophoresis profile with dermatan sulfate and heparan sulfate. MPS I was confirmed by enzymatic assay with a very low *IDUA *activity (0.1 nmol/h/mg) (usual values: 1-5). The overall physical handicap was severe and she died at the age of 11 years. In 2005, this family benefited from a prenatal diagnosis showing a normal enzymatic activity (3.5 nmol/h/mg) (usual values: 1-5).

### Patient of family F

He was the last child born in this consanguineous family after 7 MPS I unaffected brothers. By the age of 1 year, the boy was referred to Pediatric unit of Djerba Island (south of Tunisia). He was evaluated for hepatosplenomegaly, splenomegaly, and facial dysmorphism. At the age of 2 years he had 78 cm (-2 S.D.) of height and 11 kg (-1 S.D.) of weight. The biological assay showed very high urinary excretion of glycosaminoglycans (102 mg/g of creatinine) (usual values: 9-46), abnormal electrophoresis profile with dermatan sulfate and heparan sulfate and very low IDUA activity (0.1 nmol/h/mg) (usual values: 1-5).

He is presently hospitalized in the Transplant Center Unit in Tunis (North Tunisia) for bone marrow transplantation. In three months, we will expect a post transplant and we will control the IDUA activity.

## Discussion

In Tunisia, prevalences of different types of mucopolysaccharidoses have been evaluated: MPS global incidence was evaluated at 2.3/100.000 lives births in 2001 [5]. Although the covering data was the widest possible, the prevalence of the MPS were undervalued and underappreciated due to ignorance of some clinical pictures by clinicians of the Tunisian public health. A national retrospective study covering the period 1970 - 2005 concerning the incidence of mucopolysaccharidoses in Tunisia, showed that prevalence of the MPS I (Hurler, Hurler/Scheie and Scheie forms), MPS III (Sanfilippo) and MPS IVA (Morquio) was estimated at 0.63, 0.7 and 0.45 for 100,000 lives births respectively [6].

MPS I is the most common mucopolysaccharidosis worldwide, with an average incidence of about 1.7 in 100.000 live births for the severe and mild forms [7]. The incidence of MPS I in Tunisia is also high, estimated at 0.63 in 100.000 live births [6], explained by the high rate of consanguinity.

Indeed, Tunisian population is characterized by the large family size, high maternal and paternal age [2] and a particularity high level of inbreeding with consanguinity rates in the range of 32-60% (all disconcerted degree), according to a traditional practice followed the same tribe, will age or social unit [8]. These high consanguinity rates lead to the emergence of autosomal recessive disease at high frequencies; L Romdhane and al. described 346 genetics disorders identified in Tunisian population. Among these, 62.9% are autosomal recessive in which MPS I are included [2] also in Moroccan population [9].

In fact, all affected patients studied here are offspring of consanguineous marriages between first degree cousins in families 2, 4, 5, 6, C, D and E, second cousins in families 3, 7, 8, B and F and third cousins twice removed in families 1 and A (Table 1). This social fact may explain the high frequency of MPS I detection in Tunisian population.

Our molecular study [8,10-12] covering the period 2004 - 2011 showed that the most common MPS I mutation in our country are P533R which represent 53.57% of all mutated MPS I alleles found in this cases study. These data are consistent with the previous Tunisian study where three novel mutations were found [11,12]. Tunisia has different MPS I mutations frequencies (Table 2) compared to those of other European countries. We found that 53.57% (15/28) mutated alleles carried the p.P533R mutation, 52.57% of them in a homozygosity status and 1% in a heterozygosity status. Such a high prevalence of this mutation has already been described in the Maghrebian population such as in Morocco (92%) in Tunisia and also in the Maghrebian immigrant population in France [13,14]. In Europe, this mutation has only been found among Italian patients with the frequency of 11% [15].

We actually discovered that the three affected families (families 3, B and F) have a parental relationship (Figure 1), suggesting that these families possibly originated from a common ancestor. Both traditional and social concerns such as geographical proximities, similar instruction level, lead the couples to behave in a collective model, whatever conscious degree they have about the consequences.

The mucopolysaccharidoses such as MPS I and IVA have been frequently reported in studies from all over the world such as in Japan and Australia; however the impact of inbreeding in the incidence of these types of disorders has not been described despite of the important rate of consanguinity in some isolated communities such as in Hirado in Japan (14.7%) [16] or in immigrant ethnic groups, e.g., Lebanese in Australia (35.8%) [17]. An association between consanguinity and the MPS I and IVA, especially in Tunisia, is suggested according to general consanguineous data in addition to our specific Tunisian experience in MPS I and MPS IVA (pedigrees, inbreeding coefficients) [18].

It should be noted that there is a close relationship between inbreeding and not only concerning the high incidence of certain molecular lesions and their founder effect but also the appearance of new mutations e.g., p.F177S, p.L530fs and p.L578Q.

Associations between a specific mutation and a specific haplotype has been already demonstrated in genetic disorders [18] and revealed that the specific mutation was on a common background which suggests that these mutant alleles were "identical by descent" and were derived from a common ancestor.

Furthermore, the Tunisian population is also characterized by its heterogeneity in its genetic structure resulting from the mixing with other human groups from the history by invasions and migrations [3]. A large spectrum of mutations leading to some disorders such as hemoglobinopathies especially beta-thalassemia is identified as a result of rare alleles selection [3].

In fact, inbreeding increases the frequency of homozygotes in the population and hence the risk of suffering morbid. According to several studies, this behavior appears to be closely linked to socio-economic and cultural populations. Arab-Muslim populations are more affected by this practice than others. In Arab societies, all categories of cousins are getting married [2,13,14,16]. Studies of Arab and Islamic families show that inbreeding is still a common feature of the system of alliances still contracted in many countries i.e. Jordonia, Palestine, Syria, Iraq, Kuwait, Saudi Arabia, Kurdistan, Iran, Pakistan, Egypt, Sudan, North Africa (e.g. Tunisia) and Lebanon [19-21].

MPS present a social and economic impact at familial and also national level since these diseases (all types taken together and also MPS I separately) are still not rare in our area and there is no effective therapeutic means, until now. This situation justifies the need and importance of prenatal diagnosis. Two families (1 and E) received a prenatal diagnosis [22] and one patient (family F) had given bone marrow transplantation with succeed.

## Conclusion

The identification of mutations in MPS I families in Tunisia permits genetic counseling of at-risk relatives, and should facilitate molecular prenatal diagnosis in Tunisians families which are particularly large. Consanguinity is common in the Arab world and particularly in the Middle East which is largely inhabited by Arab and Muslims. The level of inbreeding is high partially due to the lack of awareness and commitment of individuals to their traditional cultural values. The high frequency of consanguineous marriages in patients with autosomal recessive disorder (e.g. MPS I) is still very important, increasing the incidence of certain lesions such as molecular p.P533R mutation, in MPS I homozygous or double heterozygous patients in Tunisia and Morocco. A multidisciplinary preventive program e.g.: public education or teaching of medical schools is needed to alert the population.

## Abbreviations

MPS: Mucopolysaccharidoes; MPS I: Mucopolysaccharidosis type I; MPS IH: Hurler syndrome; IDUA: Alpha L iduronidase; MPS IVA: mucopolysaccharidosis type IVA; GALNS: N-acetylgalactosamine-6-sulfate-sulfatase; SD: standard deviation. DPN: prenatal diagnosis.

## Competing interests

The authors declare that they have no competing interests.

## Consent

Written informed consent was obtained from the patient for publication of this case report. A copy of the written consent is available for review by the Editor-in-Chief of this journal.

## Authors' contributions

LC and SL: wrote the manuscript. LC and SK perfromed all the work (PCR, sequencing...) in the laboratory. HC, HB and MFB participated in data analysis. SF, SL and AM revised the manuscript and save final approval of the version to be published. All authors read and approved the final manuscript.
